# Accountability Accentuates Interindividual-Intergroup Discontinuity by Enforcing Parochialism

**DOI:** 10.3389/fpsyg.2015.01789

**Published:** 2015-11-25

**Authors:** Tim Wildschut, Femke van Horen, Claire Hart

**Affiliations:** ^1^Centre for Research on Self and Identity, School of Psychology, University of SouthamptonSouthampton, UK; ^2^Department of Marketing, Vrije Universiteit AmsterdamAmsterdam, Netherlands

**Keywords:** discontinuity effect, accountability, parochialism, prisoner’s dilemma, intergroup relations

## Abstract

Interindividual-intergroup discontinuity is the tendency for relations between groups to be more competitive than relations between individuals. We examined whether the discontinuity effect arises in part because group members experience normative pressure to favor the ingroup (parochialism). Building on the notion that accountability enhances normative pressure, we hypothesized that the discontinuity effect would be larger when accountability is present (compared to absent). A prisoner’s dilemma game experiment supported this prediction. Specifically, intergroup (compared to interindividual) interaction activated an injunctive ingroup-favoring norm, and accountability enhanced the influence of this norm on competitive behavior.

## Introduction

Interindividual-intergroup discontinuity refers to the tendency for relations between groups to be more competitive or less cooperative than relations between individuals (Insko et al., 2001, 2005, 2013). Most research comparing interindividual and intergroup interactions has done so in the context of experiments with mixed-motive matrix games, like the prisoner’s dilemma game (PDG). The PDG involves the interaction between two players who can each select a cooperative or competitive choice.^1^ Each player’s outcomes are determined by the combination of both players’ choices. Each player can maximize his/her outcomes by selecting the competitive choice, regardless of the choice selected by the other player. Yet, paradoxically, when both select the competitive choice, they achieve lower outcomes than they could have achieved by mutual cooperation. Most PDG experiments contrasting interindividual and intergroup interactions have supported the discontinuity effect: interacting groups are more competitive than are interacting individuals (Wildschut et al., 2003). Although the discontinuity effect has been studied predominantly in a PDG context involving participants from individualistic cultures (e.g., US, The Netherlands; Wildschut et al., 2001), it has also been documented in non-laboratory contexts (Pemberton et al., 1996), among participants from a collectivistic culture (Japan; Takemura and Yuki, 2007), in a distributive (i.e., zero-sum) multi-issue negotiation task (Loschelder and Trötschel, 2010), and in a context where the PDG matrix was substituted by a functionally equivalent set of rules governing the exchange of folded origami products (Schopler et al., 2001).

## The Role Of Parochialism In Interindividual-Intergroup

The discontinuity effect is a highly robust and multicausal phenomenon (Wildschut et al., 2007). The ingroup-favoring-norm explanation proposes that the discontinuity effect arises in part because interindividual and intergroup interactions are governed by different norms or moral codes (Cohen et al., 2006; Wildschut and Insko, 2006). On the one hand, norms for interindividual interactions emphasize fairness (Thibaut and Walker, 1975) and reciprocity (Gouldner, 1960)—a set of rules referred to as individual morality (Cohen et al., 2010). On the other hand, norms for intergroup interactions impel group members to support the ingroup at the expense of outsiders—a set of rules referred to as group morality or parochialism (Schwartz-Shea and Simmons, 1991; Baron, 2001; Wildschut and Insko, 2007).

### Historical Background

The concept of parochialism dates back millennia. In Plato’s (1891, p. 7) *The Republic*, Polemarchus defends the maxim of classical Greek morality that “justice is the art which gives good to friends and evil to enemies.” Machiavelli (1515/1952) addressed a similar message to aspiring leaders:

And yet he must not mind incurring the scandal of those vices, without which it would be difficult to save the state, for if one considers well, it will be found that some things which seem virtues would, if followed, lead to one’s ruin, and some others which appear vices result in one’s greater security and well-being (p. 93).

Hobbes (1660/1983) captured the essence of this idea in a few simple words. “Force and fraud” he wrote, “are in war the two cardinal virtues.” More recently, the theologian Niebuhr (1941) expressed a related viewpoint when he wrote:

The group is more arrogant, hypocritical, self-centered and more ruthless in the pursuit of its ends than the individual. An inevitable moral tension between individual and group morality is therefore created. … This tension is naturally most apparent in the conscience of the responsible statesmen, who are bound to feel the disparity between the canons of ordinary morality and the accepted habits of collective and political behavior (p. 222).

Early social psychological perspectives on group behavior also showed a keen awareness of group morality or parochialism. For example, Le Bon (1895/1896) wrote:

Taking the word “morality” to mean constant respect for certain social conventions, and the permanent repression of selfish impulses, it is quite evident that crowds are too impulsive and too mobile to be moral. If, however, we include in the term morality the transitory display of certain qualities such as self-abnegation, self-sacrifice, disinterestedness, devotion, and the need of equity, we may say, on the contrary, that crowds may at times exhibit a very lofty morality (p. 43).

In a similar vein, McDougall (1920) observed:

The group spirit secures that the egoistic and the altruistic tendencies of each man’s nature, instead of being in perpetual conflict, as they must be in its absence, shall harmoniously co-operate and re-enforce one another throughout a large part of the total field of human activity (p. 79).

Although influential in the very early days of social psychology, Le Bon’s (1895/1896) and McDougall’s (1920) ideas fell by the wayside after the centerpiece of their analysis—the group mind concept—was criticized by Allport (1924). As illustrated by this Research Topic, however, parochialism has recently attracted revived interest from across the social sciences (Wildschut et al., 2002; Choi and Bowles, 2007; De Dreu et al., 2014; Rusch, 2014). In the present research, we focus specifically on the contrast between norms governing interindividual interactions and the dictates of parochialism. According to the ingroup-favoring-norm explanation of the discontinuity effect, this contrast can shed light on the enduring question of why relations between groups are more competitive, hostile, and intractable than are relations between individuals.

### Empirical Evidence

Initial tests of the ingroup-favoring-norm explanation were guided by the notion that accountability enforces norms (Semin and Manstead, 1983; Tetlock, 1992; Sedikides et al., 2002). Broadly speaking, accountability is “the condition of being answerable for conducting oneself in a manner that is consistent with relevant prescriptions for how things should be” (Schlenker and Weingold, 1989, p. 24). A corollary of the norm-enforcement role of accountability is that ingroup-favoring norms should be more influential when group members are accountable rather than unaccountable to the ingroup. Only when group members are accountable can their actions influence how the ingroup evaluates them (Deutsch and Gerard, 1955). An experiment by Wildschut et al. (2002) supported this line of reasoning. Participants were placed in separate rooms and informed that they were part of a group that would interact with another group located in an adjoining laboratory. They then made individual PDG decisions under one of two conditions. In the public condition, participants were told that, upon completion of the experiment, they would meet the members of their ingroup to discuss their decisions. In the private condition, they were told that they would be dismissed separately from the laboratory. Consistent with the ingroup-favoring-norm explanation, public-condition participants (i.e., those accountable to the ingroup) made more competitive choices than did private-condition participants (i.e., those unaccountable to the ingroup). Pinter et al. (2007) conceptually replicated this finding by demonstrating that group leaders who were accountable to the ingroup made more competitive PDG choices than did unaccountable group leaders or individuals. Beyond the PDG context, Adams’s (1976) boundary role theory has stimulated research aimed at understanding how representatives react to constituent pressures in the context of intergroup bargaining. Consistent with the idea that representatives often assume that constituents expect them to be competitive toward other groups, research indicates that accountable (compared to unaccountable) representatives make fewer concessions, use more contentious tactics, and are less likely to reach agreements (Pruitt and Carnevale, 1993; Druckman, 1994).

There is, then, compelling evidence that, when group members are accountable to the ingroup, normative pressure to support the ingroup can manifest as intergroup competition. Yet, existing research is limited because it examined the effect of accountability on competitiveness in the context of intergroup interactions only. The untested assumption is that, because interindividual interactions are governed by norms of fairness and reciprocity, accountability should not increase (and might even reduce) competition between individuals, and thus accentuate the discontinuity effect. Accordingly, support for the ingroup-favoring-norm explanation is incomplete. The primary objective of the present research was to remedy this by testing the effect of accountability on competitiveness in the context of intergroup *and* interindividual interactions.

## Accountability In Interindividual Context

The effects of accountability on judgment and decision-making in interindividual contexts have been equivocal (Lerner and Tetlock, 1999). One strand of evidence supports the assumption that interindividual interactions are governed by norms of fairness and reciprocity, and, accordingly, that accountability reduces competitive behavior (Reis and Gruzen, 1976; Prentice-Dunn and Rogers, 1982). For instance, De Cremer et al. (2001) demonstrated that, in a social dilemma task, individuals who anticipated meeting their interaction partners were less competitive than those who did not anticipate such a meeting, suggesting that accountability (induced via anticipated future interaction) increased the salience of fairness norms.

Another strand of evidence suggest, however, that accountability may increase, rather than reduce, interindividual competition. Miller (1999; Miller and Ratner, 1996) proposed that, in individualistic cultures, self-interest is considered normative and rational. This norm of self-interest is both descriptive (i.e., relating to which behaviors are typically enacted) and injunctive (i.e., relating to which behaviors are typically approved or disapproved; Cialdini et al., 1990). It is descriptive in the sense that individuals believe that others’ behavior is guided by self-interest (Miller and Ratner, 1996, 1998) and it is injunctive in the sense that individuals believe others do not approve behavior that is divorced from self-interest (Ratner and Miller, 2001). Thus, accountability could enforce the norm of self-interest and attendant competition, at least in individualistic, Western cultures.

## The Present Research

The ingroup-favoring-norm explanation proposes that, whereas intergroup interactions are guided by norms impelling group members to favor the ingroup, interindividual interactions activate norms emphasizing fairness and reciprocity. Because accountability enforces norms (Tetlock, 1992), it should increase intergroup competition and reduce interindividual competition. This, in turn, entails a larger discontinuity effect in the presence (vs. absence) of accountability (Hypothesis 1). Specifically, an intergroup (compared to interindividual) context should render salient the ingroup-favoring norm, and accountability will enhance the influence of this norm on competition (Hypothesis 2). We did, however, also consider the alternative possibility that accountability enforces the norm of self-interest (rather than fairness and reciprocity) in interindividual contexts, in which case it could increase (rather than reduce) interindividual competition.

## Materials and Methods

### Participants and Design

Two hundred thirty-six female University of Southampton undergraduates took part in this experiment for partial course credit or payment (£4.00). All participants earned an additional £1.00 during the experiment. The experiment was reviewed and approved by the University of Southampton Psychology Ethics Committee. All participants provided written informed consent.

The design comprised two manipulated independent variables: interaction type (individuals vs. groups) and accountability (public vs. private responding). The interaction type variable entailed a contrast between interactions involving two isolated individuals with interactions involving two group members who belonged to two separate three-person groups. We manipulated accountability by informing participants in the public condition that, upon completion of the experiment, they would meet the two other participants seated on their side of the laboratory (henceforth, same-side others) to discuss each other’s decisions (accountability present). In the private condition, we told participants that they would be dismissed separately and that their decisions would remain anonymous (accountability absent). In the intergroup condition, we informed participants that the same-side others were part of their three-person group. In the interindividual condition, we described the same-side others as participants completing the same experiment.

### Procedure

We ran the experimental sessions in a laboratory containing six cubicles, with three cubicles located on opposite sides of the room. In the interindividual condition, these cubicles were numbered 1 through 6. In the intergroup condition, the three cubicles on one side of the room were labeled A1 through A3 and the cubicles on the other side were labeled B1 through B3. Each cubicle contained a desktop computer, a set of headphones, and a web camera. Each participant was seated in a separate cubicle. In the intergroup condition, we assigned participants to groups (A or B) and informed them that the other members of their group would be seated on the same side of the laboratory, whereas the members of the other group would be seated on the other side of the laboratory. We omitted these instructions in the interindividual condition.

Next, we explained the PDG matrix to participants. In the interindividual condition, we informed participants that they would interact with the person seated in the cubicle opposite theirs and be allowed to keep the money they earned during the experiment. We informed participants in the intergroup condition that they would interact with the member of the other group seated in the cubicle opposite theirs and that, upon completion of the experiment, the three members of their ingroup would share equally the money they had earned. In the public condition, we informed participants that, upon completion of the experiment, they would meet the same-side others to talk about the decisions that they had made. (In fact, this meeting did not occur and we dismissed participants separately.) We informed participants in the private condition that they would be dismissed separately and that their decisions would remain anonymous. Subsequently, participants completed a brief check of their understanding of the PDG and, if necessary, had their answers corrected by the experimenter.

At this point, we told participants that they would interact with the person in the opposite cubicle for one trial. This trial proceeded as follows: participants had one minute to think about the situation privately. After this 1-minute period, participants opened an audio-visual connection with the person in the opposite cubicle. Participants then had one minute to discuss the situation with the person in the opposite cubicle, whom they could hear through their headphones and see on their monitor. Following this communication period, participants had one minute to make their final decision and record it in writing. After participants recorded their decisions, the experimenter collected the decisions and distributed a post-experimental questionnaire with supplemental dependent variables. Finally, we paid all participants a standard amount of £1.00 regardless of their decisions. Debriefing followed.

### Dependent Variables

#### Manipulation Checks

To assess the effectiveness of the accountability manipulation, we asked participants: “Did you expect that the other persons seated in the cubicles on your side of the room would find out what decision you made?” (0 = *no*, 1 = *yes*). Perceived accountability should be higher with public (compared to private) responding. As a check on the interaction-type manipulation, we administered the following item: “Did you expect that every person seated in the cubicles on your side of the room would take home the same amount of money at the end of the study?” (0 = *no*, 1 = *yes*). Perceived outcome interdependence should be higher in the intergroup (compared to interindividual) condition.

#### Competitive Choice and Choice Reasons

The focal dependent variable was PDG choice behavior (0 = *cooperative*, 1 = *competitive*). Because each of the two PDG choices can be selected for a number of different reasons (e.g., the cooperative choice may reflect a concern for maximizing joint outcomes or a concern for achieving equal outcomes), we also assessed participants’ choice reasons. Participants rated 20 items, each designed to measure one of the following reasons: Max Own (e.g., “to earn as much as possible”; “to maximize my earnings”); Max Rel (e.g., “to earn more than the other person”; “to maximize the difference between the two persons in my favor”); Fear (e.g., “did not trust the other person”; “to defend myself against the other person”); Min Dif (e.g., “to minimize the difference between both persons”; “to earn an equal amount”); and Max Joint (e.g., “to earn as much as possible together”; “to maximize the joint outcomes of both persons”). Participants rated these choice reasons on 7-point scale (1 = *not at all important*, 7 = *very important*). The reliabilities for these five 4-item scales ranged from 0.72 to 0.94. We averaged the four items in each scale to create composite measures.

#### Perceived Strength of Competitive Norms

We assessed both the descriptive and injunctive aspect of perceived competitive norms. To assess the strength of the descriptive competitive norm (i.e., relating to which behaviors are typically enacted), participants estimated the number of same-side others (excluding themselves) who selected the competitive choice (range = 0–2). To assess the strength of the injunctive competitive norm (i.e., relating to which behaviors are typically approved or disapproved), participants indicated which choice they believed the same-side others wanted them to make (i.e., the choice they would approve; 0 = *cooperative*, 1 = *competitive*).

### Analysis Strategy

The experiment involved interaction between two participants, arranged in pairs. Because participants within each pair influenced each other’s responses, they cannot be treated as independent observations. Accordingly, the unit of analysis was the pair of interacting participants and we analyzed the average response across participants within pairs. PDG choice behavior was coded: 0 = *cooperative*, 1 = *competitive*. When averaged across participants within pairs, this variable could assume the values 0 (both participants cooperate), 0.5 (one cooperates and one competes), and 1 (both compete). We followed the same procedure for the manipulation checks (0 = *no*, 1 = *yes*) and for participants’ estimate of the choice same-side others wanted them to make (injunctive norm). This rendered these variables amenable to analysis of variance (ANOVA).^2^

## Results

### Manipulation Checks

We present relevant means and standard deviations in **Table 1**. As intended, an Accountability (public vs. private) × Interaction Type (individuals vs. groups) ANOVA on the accountability manipulation check revealed a significant main effect of accountability only, *F*(1,133) = 136.57, *p* < 0.001, $\eta_{p}^{2}$ = 0.50. Participants experienced stronger accountability with public (compared to private) responding. Neither the interaction-type main effect [*F*(1,133) = 0.86, *p* = 0.357, $\eta_{p}^{2}$ = 0.003] nor the Accountability × Interaction Type interaction [*F*(1,133) = 1.85, *p* = 0.177, $\eta_{p}^{2}$ = 0.01] was significant. As a check on the interaction-type manipulation, we assessed perceived outcome interdependence. As intended, an ANOVA revealed a significant interaction-type main effect, *F*(1,133) = 78.22, *p* < 0.001, $\eta_{p}^{2}$ = 0.37. Participants perceived more outcome interdependence in the intergroup (compared to interindividual) condition. Neither the accountability main effect [*F*(1,133) = 0.63, *p* = 0.430, $\eta_{p}^{2}$ = 0.003] nor the Accountability × Interaction Type interaction [*F*(1,133) = 0.09, *p* = 0.761, $\eta_{p}^{2}$ = 0.0004] was significant. In all, the accountability and interaction-type manipulations were effective.

**Table 1:** Means and standard deviations (in parentheses) for manipulation checks, competitive choice, choice reasons, and perceived competitive norms as a function of accountability (public vs. private responding) and interaction type (individuals vs. groups).

	Public		Private	
	
	Individuals	Groups	Individuals	Groups
**Manipulation checks (0–1)**				
Accountability check	0.74 (0.36)	0.86 (0.26)	0.19 (0.32)	0.17 (0.30)
Interaction-type check	0.35 (0.42)	0.89 (0.21)	0.32 (0.42)	0.83 (0.30)
Competitive choice (0–1)	0.14 (0.29)	0.50 (0.50)	0.17 (0.34)	0.26 (0.39)
**Choice reasons (1–7)**				
Max Own	3.93 (0.95)	4.35 (0.92)	4.01 (0.86)	4.09 (0.88)
Max Rel	2.33 (0.88)	3.05 (1.34)	2.41 (0.86)	2.80 (0.99)
Fear	2.44 (0.94)	2.73 (0.99)	2.46 (1.07)	2.92 (1.30)
Min Dif	5.92 (0.94)	5.30 (1.12)	5.63 (1.24)	5.42 (1.02)
Max Joint	6.07 (1.01)	5.33 (1.16)	5.89 (1.08)	5.38 (1.28)
**Perceived competitive norm**				
Descriptive norm (0–2)	0.81 (0.49)	1.18 (0.48)	0.75 (0.47)	1.14 (0.64)
Injunctive norm (0–1)	0.14 (0.29)	0.55 (0.40)	0.22 (0.35)	0.50 (0.42)


### Competitive Choice

An Accountability (public vs. private) × Interaction Type (individuals vs. groups) ANOVA on competitive choice revealed a significant main effect of interaction type, *F*(1,133) = 11.83, *p* < 0.001, $\eta_{p}^{2}$ = 0.08. Interactions between members of different groups were more competitive than interactions between individuals (i.e., a discontinuity effect). The accountability main effect was not significant, *F*(1,133) = 2.59, *p* = 0.110, $\eta_{p}^{2}$ = 0.02. The numerical pattern was for participants to be more competitive in the public (compared to private) condition. Importantly, we obtained a significant Accountability × Interaction Type interaction, *F*(1,133) = 4.28, *p* = 0.041, $\eta_{p}^{2}$ = 0.03. Tests of simple effects indicated that group members were significantly more competitive than individuals in the public condition, *F*(1,133) = 14.64, *p* < 0.001, $\eta_{p}^{2}$ = 0.10, but not in the private condition, *F*(1,133) = 0.97, *p* = 0.325, $\eta_{p}^{2}$ = 0.01. As hypothesized, the discontinuity effect was stronger with public than with private responding. Looked at in a different way, accountability significantly increased competition in the intergroup condition, *F*(1,133) = 6.72, *p* = 0.011, $\eta_{p}^{2}$ = 0.04, but not in the interindividual condition, *F*(1,133) = 0.11, *p* = 0.745, $\eta_{p}^{2}$ = 0.001.

### Choice Reasons

A series of ANOVAs on the five choice reasons resulted in significant main effects of interaction type on Max Rel, *F*(1,133) = 9.82, *p* = 0.002, $\eta_{p}^{2}$ = 0.07, Fear, *F*(1,133) = 4.13, *p* = 0.044, $\eta_{p}^{2}$ = 0.03, Max Joint, *F*(1,133) = 10.37, *p* = 0.002, $\eta_{p}^{2}$ = 0.07, and Min Dif, *F*(1,133) = 4.87, *p* = 0.029, $\eta_{p}^{2}$ = 0.04. The interaction type effect on Max Own was not significant, *F*(1,133) = 2.58, *p* = 0.111, $\eta_{p}^{2}$ = 0.02. We present relevant means and standard deviations in **Table 1**. Group members (compared to individuals) were more concerned with maximizing relative outcomes and feared their opponents more. Individuals (compared to group members) were more concerned with maximizing joint outcomes and minimizing the difference in outcomes between sides. There were no other significant effects.

### Perceived Norms

An ANOVA on the estimated number of competitive choices by same-side others (descriptive norm) revealed a significant main effect of interaction type only, *F*(1,132) = 17.91, *p* < 0.001, $\eta_{p}^{2}$ = 0.12. Participants in the intergroup (compared to interindividual) condition estimated that a greater number of same-side others would select the competitive choice (the descriptive competitive norm; **Table 1**). An ANOVA on the choice participants thought same-side others wanted them to make (injunctive norm) also revealed a significant main effect of interaction type only, *F*(1,133) = 29.47, *p* <0.001, $\eta_{p}^{2}$ = 0.18. Those in the intergroup (compared to interindividual) condition estimated that a greater number of same-side others wanted them to select the competitive choice (the injunctive competitive norm; **Table 1**). Intergroup (compared to interindividual) interactions rendered salient descriptive and injunctive competitive norms.

### Conditional Process Analyses

**FIGURE 1:**
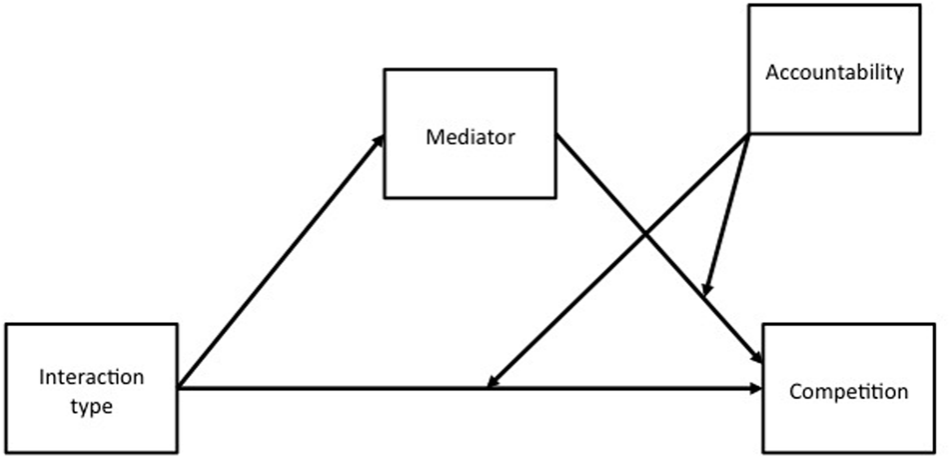
**The conditional process model tested in this experiment**.

Group members (compared to individuals) scored higher on Max Rel and Fear, and lower on Min Dif and Max Joint. Furthermore, the (descriptive and injunctive) competitive norm was stronger in the groups (compared to individuals) condition. Could any of these potential mediating mechanisms shed light on why the discontinuity effect was stronger with public (compared to private) responding? To address this question, we tested a conditional process model that Edwards and Lambert (2007) referred to as “direct effect and second stage moderation model” (see also, Baron and Kenny, 1986, p. 1179). In this model, the moderator (accountability) affects the magnitude of the mediators’ (choice reasons, perceived competitive norm) partial association with the outcome (competition) and this is found in conjunction with a main effect of the independent variable (interaction type) on the mediators (**Figure 1**). This model is appropriate because interaction type influenced the potential mediators, irrespective of accountability. Yet, interaction type influenced competition only in the public condition. We therefore tested the mediated effects of interaction type on competition, conditional upon accountability.

We first examined whether the moderator (accountability) affected the magnitude of the mediators’ (choice reasons, descriptive and injunctive competitive norms) associations with the outcome (competition) by testing, for each mediator, the Accountability × Mediator interaction. We present relevant results in **Table 2**. These analyses revealed a significant Accountability × Injunctive Norm interaction effect only (**Table 2**, A × C). Strong (compared to weak) injunctive competitive norms predicted increased competition in the public condition, *B* = 0.53, *SE* = 0.13, *F*(1,131) = 16.60, *p* < 0.001, $\eta_{p}^{2}$ = 0.10. In the private condition, the association between strength of the injunctive competitive norm and competition was not significant, *B* = 0.19, *SE* = 0.11, *F*(1,131) = 2.73, *p* = 0.101, $\eta_{p}^{2}$ = 0.02. Furthermore, the previously significant Accountability × Interaction Type interaction on competition (**Table 2**, A × B) became non-significant when we controlled for the Accountability × Injunctive Norm interaction. This indicates that the Accountability × Interaction Type interaction was “funneled through” the Accountability × Injunctive Norm interaction (Baron and Kenny, 1986, p. 1179). Accountability did not significantly moderate the association of any other mediator with competition (**Table 2**, A × C row).

**Table 2:** Conditional process analyses: testing the effect of accountabilty on the magnitude of the mediators’ association with competition (Effect A × C).

							Mediator
	
	Max Rel		Fear		Min Dif		Max Joint		Descriptive competitive norm		Injunctive competitive norm	
						
	*F*	*p*	*F*	*p*	*F*	*P*	*F*	*p*	*F*	*p*	*F*	*p*
Accountability (A)	4.90	0.029	3.87	0.051	4.87	0.029	5.95	0.016	2.30	0.132	3.33	0.070
Interaction type (B)	2.36	0.127	7.94	0.006	6.22	0.014	2.42	0.122	4.11	0.045	1.79	0.183
A × B	3.18	0.077	5.40	0.022	2.38	0.126	3.04	0.084	4.12	0.045	0.62	0.434
Mediator (C)	29.75	<0.001	21.48	<0.001	56.00	<0.001	106.07	<0.001	13.28	<0.001	17.25	<0.001
A × C	0.01	0.903	0.08	0.773	0.67	0.416	2.13	0.146	0.00	0.972	3.92	0.049


*The dependent variable in each analysis is competitive choice. Denominator degrees of freedom equal 131. For analyses involving the descriptive competitive norm, denominator degrees of freedom equal 130 due to one missing value.*

As a final step, we used the PROCESS macro to test the conditional process model depicted in **Figure 1**, with the injunctive competitive norm as mediator (model 15; 10,000 resamples; Hayes, 2013). PROCESS calculates bootstrap confidence intervals (CIs) for the indirect effect (denoted as *ab*) of interaction type on competition via a mediator (here, injunctive competitive norm), conditional upon accountability. In the public condition, this indirect effect was positive and significant (i.e., the 95% CI did not include 0), *ab* = 0.18, *SE* = 0.06, 95% CI = 0.08/0.32. In the private condition, this indirect effect was non-significant, *ab* = 0.06, *SE* = 0.04, 95% CI = -0.004/0.17. In all, the discontinuity effect was mediated by an injunctive competitive norm when accountability was present (public condition) but not when it was absent (private condition). That is, the intergroup (compared to interindividual) context strengthened the injunctive competitive norm, and accountability enforced this norm.^3^

## Discussion

According to the ingroup-favoring-norm explanation, the discontinuity effect arises in part because interindividual and intergroup interactions are governed by different norms or moral codes (Wildschut and Insko, 2006, 2007). Whereas interindividual interactions activate norms emphasizing fairness and reciprocity, intergroup interactions are guided by norms dictating ingroup-favoritism or parochialism. Because accountability enforces norms (Tetlock, 1992), the ingroup-favoring-norm explanation entails a larger discontinuity effect when accountability is present compared to when it is absent (Hypothesis 1). Results supported this first hypothesis. To be precise, when participants were accountable to others seated on their side of the laboratory (same-side others), intergroup interactions were significantly more competitive than interindividual interactions (the discontinuity effect). In the absence of such accountability, the discontinuity effect was not significant. This latter finding suggests that being part of a three-person group that shares earnings (i.e., outcome interdependence) *per se* may not be sufficient to induce the discontinuity effect. Although outcome interdependence renders salient the injunctive ingroup-favoring norm, accountability is required to enforce this norm.

Examining our findings from a different angle, we found that accountability increased intergroup competition, replicating prior research in PDG (Wildschut et al., 2002; Pinter et al., 2007) and bargaining (Pruitt and Carnevale, 1993) contexts. Matters were more complex in the interindividual context. Based on the notion that interindividual interactions are guided by norms of fairness and reciprocity (Cohen et al., 2010), we predicted that accountability would reduce interindividual competition. However, we also considered the alternative possibility that accountability could enforce a norm of self-interest (Ratner and Miller, 2001), thereby increasing competition. Results revealed that accountability neither decreased nor increased interindividual competition and, thus, neither prediction received support. This null finding could indicate that both predictions are correct and cancel-out each other. That is, in interindividual contexts, accountability may enforce norms of fairness and equality, as well as the opposing norm of self-interest. This is what McDougall (1920, p. 79) may have had in mind when he wrote that, in the absence of a “group spirit,” the “egoistic and the altruistic tendencies of each man’s nature [are in] in perpetual conflict.” Future research could examine how different individuals weigh these contrasting tendencies. Perhaps the norm of self-interest is more salient to high-narcissists, who value agency over communion, whereas norms of fairness and reciprocity are more salient to low-narcissists, who value communion over agency (Horton and Sedikides, 2009; Hart et al., 2011). If so, accountability should increase interindividual competition among high-narcissists but reduce it among low-narcissists.

Another possible explanation for the absence of a significant accountability effect in the interindividual context is that the accountability manipulation was less impactful there. The manipulation check data indicate that, regardless of interaction type, participants in the public (compared to private) condition expected their decisions to be identified by own-side others. However, we cannot rule out the possibility that the accountability manipulation had less impact on the subjective sense of accountability to own-side others in the interindividual (compared to intergroup) context. This is an important issue to consider in future research.

The conditional process analyses shed additional light on the role of accountability in interindividual and intergroup contexts. We hypothesized that an intergroup (compared to interindividual) context would render salient the ingroup-favoring norm, and that accountability would enhance the impact of this norm on competitive behavior (Hypothesis 2). Supporting this second hypothesis, participants in the intergroup (compared to interindividual) condition estimated that a greater number of same-side others would select the competitive choice (the descriptive competitive norm) and wanted them to select the competitive choice (the injunctive competitive norm). In addition, accountability strengthened the positive association between the injunctive (but not descriptive) competitive norm and competitive behavior. As a result, the discontinuity effect was mediated by an injunctive competitive norm when accountability was present but not when it was absent. The finding that accountability enforced injunctive norms only is consistent with the idea that (a) injunctive (but not descriptive) norms relate to how behaviors are typically approved or disapproved (Cialdini et al., 1990) and (b) only when group members are accountable can their actions influence how they are evaluated by the ingroup (Deutsch and Gerard, 1955).

The conditional process analyses yielded no evidence that accountability bolstered the link between choice reasons and actual choice. Group members reported more concern with maximizing relative outcomes and fear than did individuals. Concern for maximizing relative outcomes and fear, in turn, predicted increased competition irrespective of accountability. Individuals reported more concern with maximizing joint outcomes and minimizing differences than did group members. In turn, concern for maximizing joint outcomes and minimizing differences predicted reduced competition irrespective of accountability (**Table 2**). Note that, even in the public condition, participants’ stated choice reasons remained private. We think it is plausible that accountability would strengthen the association between publicly stated choice reasons and behavior because (a) actors whose publicly stated reasons are inconsistent with their behavior (e.g., stating that one wishes to maximize joint outcomes but selecting a competitive choice) would be seen as hypocritical (Barden et al., 2005; Alicke et al., 2013) and (b) such consistency (vs. inconsistency) can only be assessed when accountability is present (i.e., in the public condition). This is another avenue for future research.

### Broader Implications

Although these findings provide evidence for the postulated ingroup-favoring norm, one could argue that when a person influences the welfare of other group members and is accountable to them, it is simply rational to take their interests into account to gain their approval and avoid sanctions. Relevant to this point, Thibaut and Kelley (1959) proposed that norms arise from rationality. They illustrated this idea with an example of a husband and wife who like to go out together on weekends. Unfortunately, the wife prefers to go dancing, whereas the husband prefers to go to the movies. Thibaut and Kelley (1959) suggested that the couple can resolve this conflict of interest and maximize joint outcomes over time by alternating between jointly going to the movies on 1 weekend and jointly going dancing on the next weekend. What is a rational solution at first may then become normative over time, and hence, rationality and norms may become confounded. Thibaut and Kelley’s (1959) general argument is compatible with Bentham’s (1789/1879) and Mill’s (1863) concept of utilitarianism—that norms arise from what is the greatest good for the greatest number.

The concept of an ingroup-favoring norm may also shed light on the question of how individual preferences are combined to reach group decisions. Using a social decision scheme approach, Morgan and Tindale (2002) examined social influence processes within three-person groups by asking group members to make individual PDG choices before engaging in a discussion to reach consensus regarding a group decision. They found that when the individual preferences indicated unanimity among the three group members, the final group decision almost always corresponded to these individual preferences. When the group members’ individual decisions were not unanimous, however, an interesting asymmetry occurred. Whereas a competitive group decision was reached in 91% of cases when all but one group member had initially indicated a competitive preference, a cooperative group choice was only reached in 48% of cases when all but one group member had initially indicated a cooperative preference. That is, whereas majorities favoring competition were rarely persuaded to change their view, majorities favoring cooperation were persuaded to change their view in most cases. Morgan and Tindale (2002, p. 49) interpreted these asymmetric social influence patterns in terms of shared task representations or “any task/situation relevant concept, norm, perspective, processing goal, or strategy that is shared by most or all of the group members.” They proposed that when arguments are stated that are consistent with a shared task representation, even majority members can be influenced to change their initial position. We think that the ingroup-favoring norm is central to group members’ shared task representation when there is a conflict of interest with an out-group.

### Limitations and Future Directions

Before generalizing from these findings, it is important to keep in mind that the sample consisted exclusively of Western, female undergraduates. The question whether culture has a bearing on the role of accountability in interindividual and intergroup contexts presents a fruitful direction for future research. A primary dimension on which cultures and their members can be differentiated is individualism-collectivism (Hofstede, 1980). In individualistic cultures (such as the UK, where we conducted the present experiment), the independent, agentic self predominates. In collectivist cultures, the interdependent, communal self predominates (Triandis, 1989). Gelfand and Realo (1999) showed that, in the context of intergroup bargaining, accountability increased competition between group representatives with low levels of collectivism (as in the present experiment with UK participants) but increased cooperation between those with high levels of collectivism. Their findings suggest that the catalytic effect of accountability on interindividual-intergroup discontinuity may be stronger in individualistic than collectivistic cultures.

Nonetheless, there are important differences between the PDG and the tasks employed by Gelfand and Realo (1999). They investigated a combination of distributive (i.e., zero-sum) and integrative bargaining scenarios. Schopler et al. (2001) proposed that, in zero-sum situations, there is no one choice that benefits both players. Because this is true for interactions between groups and interactions between individuals, there is no reason to expect a discontinuity effect in a distributive bargaining context. They further noted that, when an integrative solution is available, mutual cooperation benefits both players more than mutual competition. Because this is true for relations between groups and relations between individuals, there is no reason to expect a discontinuity effect in an integrative bargaining context either. Consistent with these arguments, Schopler et al. (2001) demonstrated that the discontinuity effect arises when, as in the PDG, mutual cooperation benefits both players whereas competition benefits one player over the other (also see Wildschut et al., 2003). Whether Gelfand and Realo’s (1999) evidence for the moderating role of individualism-collectivism generalizes to a PDG context is an important question for future research.

Finally, we recruited exclusively female participants because (a) we decided to limit our experiment to same-gender interactions (to eliminate gender composition of experimental sessions as a source of random error) and (b) females vastly outnumber males in our participant pool (∼8:1). To the best of our knowledge, there is no systematic evidence to suggest that gender moderates the effect of accountability in either interindividual or intergroup contexts. Nonetheless, future research on this topic would do well to study both males and females.

### Coda

The present findings add to our understanding of why intergroup relations are often more antagonistic and violent than are interindividual relations: accountability enforces parochialism in intergroup contexts. We hope that these and other advances will provide a basis for effective interventions aimed at promoting intergroup cooperation and harmony.

## Conflict of Interest Statement

The authors declare that the research was conducted in the absence of any commercial or financial relationships that could be construed as a potential conflict of interest.

## Footnotes

1 In this article, we refer to the two PDG choices as cooperative choice and competitive choice, respectively. Elsewhere, the competitive choice is also referred to as the non-cooperative choice or defection. The latter labels have the advantage of not suggesting relativistic motivation or a concern with winning. However, we used the “competitive choice” label for ease of exposition and to maintain consistency with previous work on the discontinuity effect (e.g., Insko et al., 2013). By adopting this terminology, we do not mean to suggest that the competitive choice necessarily reflects concern with winning.

2 To demonstrate that the results for the dichotomous variables (i.e., manipulation checks, competition, and injunctive norm) are not purely an artifact of our preferred ANOVA strategy, we conducted alternative analyses, using SAS PROC GLIMMIX. In these logistic analyses, the individual participants (rather than interacting pairs) were treated as units of analysis. To control for the dependence between participants within each pair, we included pairs as a random variable in a multilevel model. We specified that the dependent variables have a binary distribution. The pattern of significant (and non-significant) results was identical, with one minor exception: the Accountability × Interaction Type interaction on competition was significant in the ANOVA (see below) and marginal in the multilevel logistic analysis, χ^2^(1) = 2.97, *p* = 0.085.

3 We also conducted a parallel mediation analysis, in which we included simultaneously all six mediators for which the interaction type main effect was significant (i.e., descriptive and injunctive competitive norm, Max Rel, Fear, Min Dif, and Max Joint). In this analysis, the Accountability × Injunctive Norm interaction remained significant, *F*(1,120) = 4.19, *p* = 0.043. Accountability did not significantly moderate the partial association of any other mediator with competition (*p*s > 0.076). In the parallel mediation analysis, the indirect effect of interaction type on competition via the injunctive competitive norm became marginal in the public condition (*ab* = 0.08, *SE* = 0.05, 90% CI = 0.01/0.19) and remained non-significant in the private condition (*ab* = -0.03, *SE* = 0.04, 90% CI = -0.09/0.03). We suspect that the slightly weaker evidence for the injunctive norm in these parallel mediation analyses is due to the inclusion of multiple correlated mediators and resultant multicollinearity.
